# Immortalization of Mesenchymal Stem Cell Lines from Sheep Umbilical Cord Tissue

**DOI:** 10.3390/biology13070551

**Published:** 2024-07-22

**Authors:** Jinwei Yang, Yitong Dong, Lixinyi Hu, Weihai Wang, Yajun Li, Shujie Wang, Chunsheng Wang

**Affiliations:** 1 College of Life Science, Northeast Forestry University, Harbin 150040, China; 2 State Key Laboratory for Animal Disease Control and Prevention, Harbin Veterinary Research Institute, Chinese Academy of Agricultural Sciences, Harbin 150001, China

**Keywords:** sheep, immortalization, mesenchymal stem cells, TERT, differentiation

## Abstract

**Simple Summary:**

Simple Summary: Mesenchymal stem cells (MSCs) are adult stem cells capable of differentiating into various cell types, including osteoblasts, chondrocytes, and adipocytes. Renowned for their immunomodulatory and regenerative properties, MSCs hold great promise as tools for tissue repair and therapeutic applications. To facilitate the large-scale production of sheep umbilical cord MSCs (UCMSCs), the telomerase reverse transcriptase (TERT) gene was introduced into UCMSCs to promote immortalization. This study provides valuable insights into (a) the proliferation and anti-aging capabilities of transgenic MSCs compared to primary MSCs; (b) the absence of potential tumorigenicity exhibited in TERT-UCMSCs; and (c) the differentiation potential of TERT-UCMSCs. Immortalized MSCs exhibit enhanced proliferation and anti-aging abilities without tumorigenicity concerns, making them a safe immortalized cell line suitable for medical treatments.

**Abstract:**

Mesenchymal stem cells (MSCs) possess significant differentiation potential, making them highly promising in medicine and immunotherapy due to their regenerative capabilities and exosome secretion. However, challenges such as limited cell divisions and complex testing hinder large-scale MSC production. In this study, we successfully established an immortalized MSC line by transfecting the human telomerase reverse transcriptase (TERT) gene into MSCs isolated from pregnant sheep umbilical cords. This approach effectively inhibits cell senescence and promotes cell proliferation, enabling the generation of umbilical cord mesenchymal stem cells (UCMSCs) on a larger scale. Our findings demonstrate that these transfected TERT-UCMSCs exhibit enhanced proliferative capacity and a reduced aging rate compared to regular UCMSCs while maintaining their stemness without tumorigenicity concerns. Consequently, they hold great potential for medical applications requiring large quantities of functional MSCs.

## 1. Introduction

Mesenchymal stem cells (MSCs) are derived from mesoderm with strong differentiation abilities. Due to their characteristics of proliferation, differentiation, regeneration, immune regulation, and exosome secretion [1], MSCs hold broad promises for applications in medicine [2,3], beauty [4], and the treatment of diseases resistant to conventional drugs [5,6].

MSCs are plentiful and can be sourced from bone marrow [7], adult peripheral blood [8], umbilical cord [9], and adipose tissue [10]. Notably, they exhibit low immunogenicity [11], minimizing harm to the recipient, and are considered ideal for tissue repair [12]. Although originally isolated from bone marrow [13], the process is complex and often associated with discomfort. Hence, MSCs derived from discarded umbilical cord tissue serve as a preferable source.

As an extra-embryonic tissue, the umbilical cord can be obtained without causing harm to the organism, thereby reducing both maternal discomfort and cost. Umbilical cord mesenchymal stem cells (UCMSCs) derived from Wharton’s jelly have been reported to possess the capacity for osteogenic, lipogenic, and chondrogenic differentiation under appropriate conditions [14] and have been utilized in promoting articular cartilage injury repair [15]. As a shared component of both the fetus and maternal, the placenta may harbor maternal cells; hence, relying on a single cell source is insufficient for research material. Peripheral blood, although similar to umbilical cords as a source of MSCs, has a low MSC content. Thereby, the umbilical cord is a more suitable source to isolate MSCs.

Umbilical cord-derived MSCs from sheep find utility in regenerative medicine, animal tissue engineering [16], various cell experiments, and clinical treatments [17,18], such as ameliorating acute renal failure (ARF) [19], treating intervertebral disc degeneration (IVD) [20], and alleviating osteoarthritis in sheep [21].

However, the application of MSCs is hindered by their limited passage times. This limitation is primarily due to low telomerase activity. To address this, the long-term passage of MSCs can be achieved by introducing virus genes like human papillomavirus (HPV) [22], Simian Virus 40 (SV40) [23], transferring the human telomerase gene [24], or transfecting oncogenes [25]. Notably, the telomerase reverse transcriptase (TERT) gene provides a safer and more suitable solution, enhancing endogenous telomerase activity and extending cell lifespan, thereby increasing the passage number [26].

The potential tumorigenic risk associated with MSCs has drawn significant concerns. Immortalization technologies may compromise genetic stability, leading to uncontrolled proliferation and malignant transformation of MSCs [27], potentially resulting in tumor formation. Intracellular gene mutations [28], chromosomal abnormalities [29], and epigenetic modifications [30] can drive cells towards malignancy and increase tumorigenic risk. Furthermore, TERT gene transfection may contribute to tumor formation [31], posing a serious threat to patient safety. Hence, understanding and researching the safety profile of immortalized MSCs is critical.

This study aimed to expand sheep umbilical cord MSCs production by increasing the passage number to establish an immortalized sheep MSCs line. Various properties of sheep MSCs were investigated during this process, including proliferation capacity, differentiation potential, anti-aging characteristics, and tumorigenicity.

## 2. Materials and Methods

### 2.1. Ethic Statement

All animal experimental procedures were approved by the Animal Ethics Committee of Northeast Forestry University (protocol code 2021001, November 2020) and conducted in accordance with the requirements of SPF-grade experimental animal facilities.

### 2.2. Animals

Umbilical cord tissue was purchased from a slaughterhouse (northeast fine wool sheep, 3 years old, 145 days pregnant). The ends of the sheep’s umbilical cords were tied with sterilized sutures immediately after birth. Umbilical cords, 10–15 cm in length, were then cut with scissors and transported promptly to the stem cell laboratory. Upon arrival in the laboratory, the tissue was sterilized. After disinfection, the umbilical cord was transferred to a biosafety cabinet (ESCO, Model: AC2-4S1, ESCO, Beijing, China) and soaked in PBS with antibiotics. They were then washed three times with PBS. The amniotic membrane was carefully unfolded, and the two veins and one artery within the umbilical cord were removed using a hemostatic clamp. Following another three washes with PBS, the Wharton’s jelly covering the blood vessels was scraped from the amniotic membrane using a blade. Subsequently, the tissue was cut into pieces measuring 0.5–1 mm^3^ with surgical scissors.

### 2.3. Stem Cell Isolation and Culture Expansion

Stem cells were collected from the Wharton’s jelly of sheep umbilical cord tissue. The Wharton’s jelly was placed in a 10 cm petri dish containing 4 mL of base medium and cultured in an incubator at 37 °C with 5% CO_2_ (SANYO, Model: MCO-18AIC, SANYO, Shanghai, China). Tissue adhesion was observed 48–72 h later, and the medium was changed every three days. When cell confluence reached 70%, the tissue blocks were washed with PBS, collected in a centrifuge tube, and centrifuged at room temperature at 1200 rpm for 4 min. The supernatant was discarded, and the cells were suspended in the medium. The cells were then re-inoculated into a 10 cm petri dish with 4 mL of culture medium, which was replaced with 10 mL of culture medium once the cells were fully attached. The medium was replaced every 3 days. When cell confluence reached 90%, the cells were passaged using trypsin digestion for 2 min at 37 °C in a 5% CO_2_ environment, with the medium changed every 3 days.

### 2.4. TERT Cotransfection

The recombinant plasmid pLV2-CMV-TERT (human)-IRES-Puro (P37541) was obtained from the Miaoling Plasmid Platform. *Escherichia coli* was used for large-scale culture expansion to amplify the plasmids, and the EndoFree Maxi Plasmid Kit (Cat. DP117) from Tiangen^®^ (Beijing, China) was employed to extract endotoxin-free plasmid. HEK293T cells were revived in a culture dish and used for experimentation once they reached a cell density of 50% to 60%. A mixture containing 6 μg of pLV2-CMV-TERT (human)-IRES-Puro (P37541), 4.5 μg of psPAX2, and 1.5 μg of pMD2.G diluted in 500 μL of basal medium was prepared. The original medium was replaced with 2.5 mL of basal medium supplemented with 15 μg of PEI. The viral plasmid mixture was added to the cells and incubated at 37 °C for 24 h. The cells were then incubated with a basal medium for 48 h. The infection process was repeated, and after another 24 h, the supernatant was collected.

### 2.5. Infection and Screening

The viral supernatant obtained previously was filtered using a 0.45 μm membrane filter, and the culture medium of UCMSCs was replaced with the filtrate. Polybrane at a concentration of 5 μg/mL was added and then incubated at 37 °C for 24 h. The medium was changed to a complete medium, and a concentration of 1 μg/mL puromycin was added. Meanwhile, a control group without puromycin was established. After culturing for several days until the control group cells almost disappeared, cell viability was assessed under a microscope and the screening culture of the infected group was continued for 2 days. The cultures were subsequently expanded and cryopreserved for future experimental use.

### 2.6. Surface Marker Expression Analysis

The expression of surface markers (CD14, CD34, CD44, CD45, CD105) of sheep umbilical mesenchymal stem cells was analyzed using flow cytometry. UCMSCs were cultured at 37 °C in a 5% CO_2_ incubator until the fusion reached 90%, washed with 1×PBS 3 times, and EDTA-0.25% Try was used to digest the adherent cells. At room temperature, centrifuge at 1000 rpm for 5 min and discard the supernatant. Add 1 mL 1×PBS for re-suspension and wash twice, centrifuge for 1000 rpm 5 min each time, and discard the supernatant. The 200 µL flow cell buffer was re-suspended in a 1.5 mL centrifuge tube with a density of 1 × 10^6^ cells per 100 µL and placed at room temperature for 30 min. Primary antibody (1:500) was added and incubated at 37 °C for 1 h away from light (The following steps are required to avoid light operation). Centrifuge at room temperature for 1000 rpm 5 min, discard the supernatant, and add 1×PBS. Centrifuge at 1000 rpm for 5 min and discard the supernatant. The secondary antibody diluted with 100 µL 1×PBS 1:200 was added and incubated at 37 °C away from light for 1 h. Centrifuge with 1×PBS 5000 rpm for 5 min, rinse once, re-suspend with 100 µL 1×PBS, and transfer to 2 mL centrifuge tube for analysis by flow cytometry. The antibody CD44 (Cat. AF0105) was from Beyotime (Shanghai, China). CD14 (Cat. 17000-1-AP), CD34 (Cat. 14486-1-AP), CD45 (Cat. 20103-1-AP), CD105 (Cat. 67075-1-Ig), and the secondary antibody are from Proteintech (Wuhan, China; Cat. SA00003-2 for anti-rabbit and Cat. SA00003-11 for anti-rat). The flow cytometer used was CytoFLEX (model: A00-A-1102) from Beckman Coulter (Brea, CA, USA). CytExpert software (version 2.5.0.77) was used for experimental data recording. 

### 2.7. β-Galactosidase Activity Assay

UCMSCs at passages 3 and 10 and TERT-UCMSCs at passage 30 were cultured and subjected to staining using the Cell Senescence β-Galactosidase Staining Kit (Cat. C0602) from Beyotime (Shanghai, China). Microscopic analysis was performed.

### 2.8. Quantitative PCR

Total RNA was extracted using Trizol reagent, and a first-strand cDNA synthesis was performed using HiFiScript gDNA Removal RT MasterMix (Cat. CW2020M) from CWBio (Beijing, China). The synthesized cDNA was stored at −20 °C until further use. A 20 μL reaction mixture containing MagicSYBR Mixture (Cat. CW3008M) from CWBio was prepared. The expression levels of the target gene TERT and the housekeeping gene GAPDH were detected using a Thermo Cycler (LightCycler^®^ 480 Instrument II) from Roche (Basel, Switzerland). Relative RNA levels were calculated using the 2^−ΔΔCT^ method. Primer sequences are provided in Table 1.

### 2.9. Growth Kinetics and Population Doubling Time (PDT)

Single-cell suspensions of primary UCMSCs, TERT-UCMSCs P10, P20, and P30 were prepared, seeding them at a concentration of 1000 cells per well in 96-well plates with 100 µL of medium added to each well. The plates were then incubated at 37 °C in a humidified atmosphere containing 5% CO_2_. After 24 h, the medium was replaced once. Subsequently, the cells in the wells were counted using a hemocytometer plate every 24 h. The growth kinetics curve and population doubling time (PDT) were determined based on the measured cell numbers.

### 2.10. EdU-Azide 488 and Hoechst 33342/PI Staining

After overnight culture, UCMSCs P3, MSCs P10, and TERT-MSCs P30 were restored to their normal state. Dead cells were identified using propidium iodide (PI) staining. Nuclei were stained with Hoechst 33,342 from BeyoClick™ EdU-488 Cell Proliferation Assay Kit (Cat. C0071S) of Beyotime (Shanghai, China) and subsequently visualized using fluorescence microscopy (Leica, Leica Microsystems CMS GmbH Ernst-Leitz-Str. 17–37 35,578 Wetzlar, Germany). For flow cytometry, PI was used instead of Hoechst 33342.

### 2.11. Tri-Lineage Differentiation

Mesenchymal stem cells derived from ewe umbilical cords were transfected with the TERT gene and utilized for trilineage differentiation.

#### 2.11.1. Osteogenic Differentiation

The OriCell^®^ (Guangzhou, China) Osteogenic Differentiation Kit (Cat. HUXUC-90021) was used to prepare the osteogenic differentiation medium (Table S1). Before inoculation, a 0.1% gelatin coating was applied to six-well plates by evenly distributing 1 mL of the solution at the bottom of each well. After incubation at 37 °C for 30 min and the removal of excess liquid, cultures were conducted following kit instructions. The medium was changed every 3 days. After 21 days, calcium nodules were stained with alizarin red and observed.

#### 2.11.2. Adipogenic Differentiation

The OriCell^®^ Adipogenic Differentiation Kit (Cat. HUXUC-90031) was utilized to prepare medium A and B (Table S2). Gelatin-coated dishes were employed and cultured following the protocol. Subsequently, oil red O staining was employed to visualize lipid droplets once they reached an optimal size and abundance, and microscopic analysis was performed for lipogenic staining evaluation.

#### 2.11.3. Chondrogenic Differentiation

The complete chondrogenic differentiation medium (Table S3) was prepared using the OriCell^®^ chondrogenic differentiation kit (Cat. HUXUC-90041). Gelatin-coated six-well plates were utilized following the chondrogenic differentiation protocol. The complete medium for chondrogenic differentiation was refreshed every 2 days during a 28-day induction period, and Alcian blue dye working solution was used to stain cartilage balls for observation under a microscope.

### 2.12. Soft Agar Colony Formation

The 0.7% and 1.2% low-melting point agarose solutions were prepared, autoclaved, and stored at 4 °C until further use. On the day of the experiment, the gel-like agarose was heated in a water bath at 90 °C until it completely melted into a liquid state. The lower gel mixture consisting of complete medium mixed with an equal volume of 1.2% low-melting agarose was prepared and evenly spread at a volume of 1.5 mL per well in a 6-well plate, ensuring the removal of any small bubbles. The mixture was then allowed to solidify at room temperature for 30 min. UCMSCs P3, TERT-UCMSCs P30, and human hepatoma cells (HepG2) were digested and counted to achieve a concentration of 5000 cells/well. For the upper layer gel mixture, a complete medium mixed with an equal volume of 0.7% low-melting point agarose was prepared and evenly spread at a volume of 2 mL per well in the same set of plates as before. After thorough mixing with suspension containing 5000 cells, the plates were placed in an incubator set to maintain conditions suitable for solidification (37 °C). Following incubation for approximately half an hour, each well received an additional 0.5 mL complete medium to prevent evaporation. The plates were then kept inside a cell incubator maintained at standard conditions (37 °C with 5% CO_2_) for about two weeks while regularly monitoring until signs indicating plate closure became evident.

Colony growth and formation were observed under a magnification power provided by using an objective lens set to 5×; six random fields within each plate were selected for statistical analysis.

### 2.13. Statistics Analysis

The statistical analysis was performed using GraphPad Prism 9.5.0 and FlowJo version 10.8.1 software. Student’s *t*-test was employed to compare the two groups, while one-way analysis of variance (ANOVA) was used for comparing data among multiple groups. The significance level was set at * *p* < 0.05 and ** *p* < 0.01, and a higher level of significance at *** *p* < 0.001 was considered extremely significant.

## 3. Results

**Figure 1:**
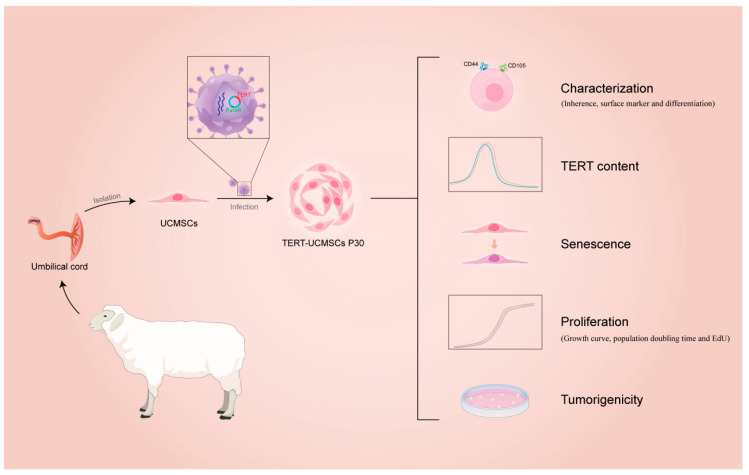
Flow diagram of immortalization of UCMSCs from sheep.

This study elucidated and compared the characteristics of TERT-transfected and non-transfected MSCs from sheep umbilical cord tissue. The flow diagram is shown in Figure 1.

### 3.1. Umbilical Cord Tissue Isolation and Culture Expansion

**Figure 2:**
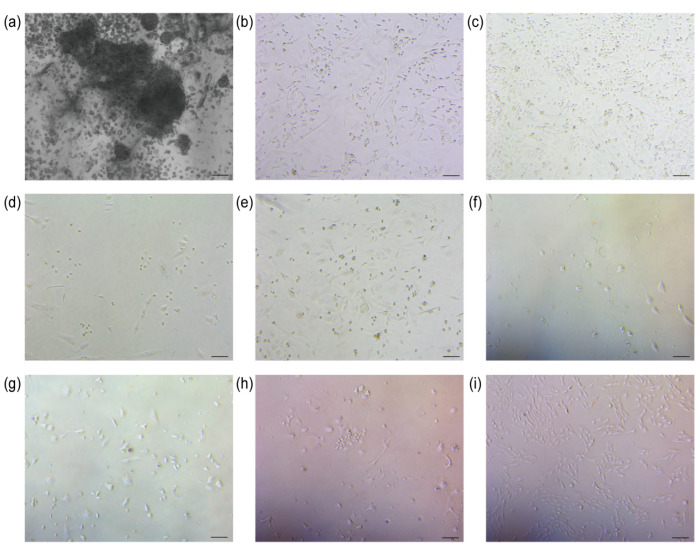
Cell isolation and screening results. (**a**) Isolation of UCMSCs (scale bar = 200 μm); (**b**) Control group 0 h of addition of puromycin (scale bar = 100 μm); (**c**) TERT-UCMSCs 0 h of addition of puromycin (scale bar = 100 μm); (**d**) Control group 24 h of addition of puromycin (scale bar = 100 μm); (**e**) TERT-UCMSCs 24 h of addition of puromycin (scale bar = 100 μm); (**f**) Control group 48 h of addition of puromycin (scale bar = 100 μm); (**g**) TERT-UCMSCs 48 h of addition of puromycin (scale bar = 100 μm); (**h**) TERT-UCMSCs 6 d of addition of puromycin (scale bar = 100 μm); (**i**) TERT-UCMSCs 10 d of addition of puromycin (scale bar = 100 μm).

The sheep’s umbilical cord tissue was meticulously processed to ensure optimal treatment. Subsequently, umbilical cord mesenchymal stem cells were successfully isolated from Wharton’s jelly under controlled conditions at 37 °C and 5% CO_2_ (Figure 2a).

### 3.2. Screening

Following the transfection of UCMSCs with the TERT gene, puromycin was used to select cells successfully transfected with the plasmid. The control group consisted of cells without lentivirus infection. Upon the addition of puromycin, the cell density in both experimental and control groups was comparable. After 24 h, the cell survival rate in the control group was approximately 10%, whereas it reached around 70% in the experimental group. After 48 h, no viable cells were observed in the control group, while 40% of cells remained viable in the experimental group (*n* = 3). Four days after completion of the screening, TERT-UCMSCs were regrown (Figure 2b–i).

### 3.3. Phenotypic Gene Expression

**Figure 3:**
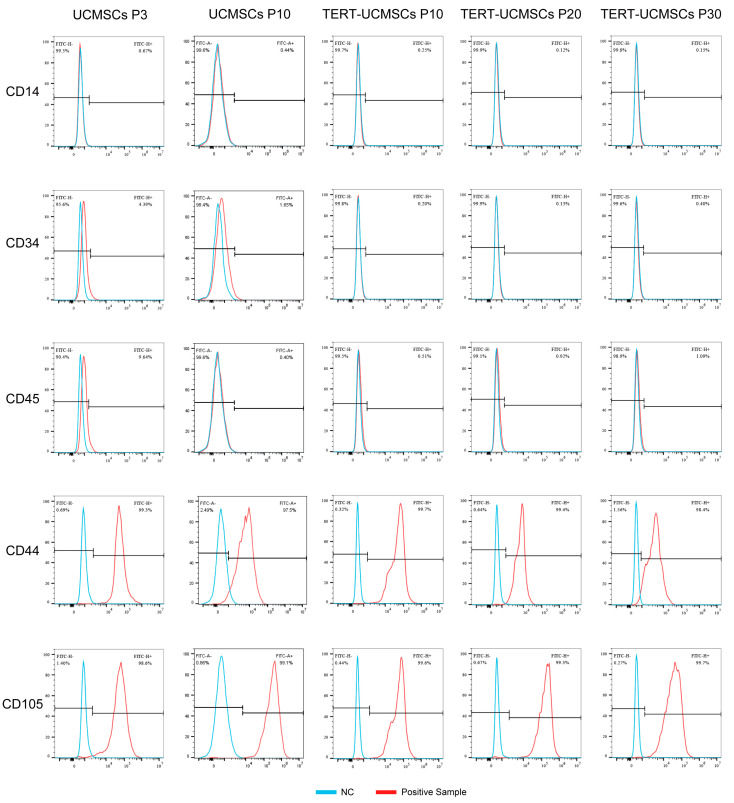
Cell surface marker results (CD14, CD34, CD45, CD44, and CD105) obtained by flow cytometry.

UCMSCs and TERT-UCMSCs showed typical characteristics for all measured surface markers. For instance, CD44 and CD105 showed high expression levels on the cell surface (above 95%), whereas CD14, CD34, and CD45 exhibited low expression levels less than 5% (Table 2, Figure 3). The relatively high percentage of CD45 in primary cells may be attributed to insufficient cell purity.

### 3.4. β-Galactosidase Activity Assay

**Figure 4:**
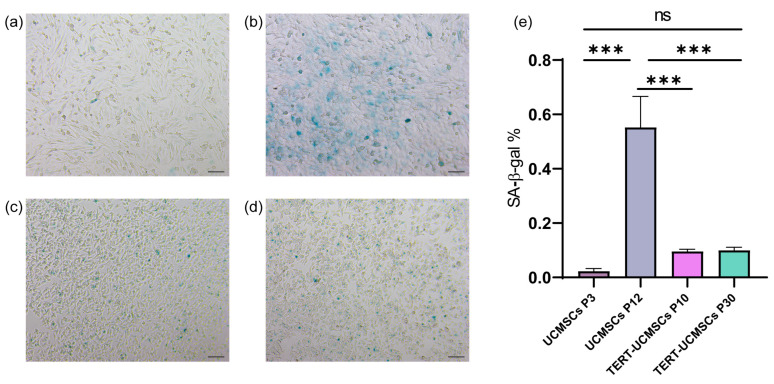
Result of β-galactosidase assay: (**a**) UCMSCs P3; (**b**) UCMSCs P12; (**c**) TERT-UCMSCs P10; (**d**) TERT-UCMSCs P30 (*n* = 3); (**e**) Percentage of SA-β-Gal positive cells in different passages of UCMSCs P3, P12, and TERT-UCMSCs P30. Scale bar = 100 μm, *n* = 3, *** *p* < 0.001, ns indicates no statistical significance.

Blue-stained spots, attributed to specific staining of β-galactosidase released by cellular senescence, were clearly observed in all three cell types. The staining percentage in UCMSCs at passage 10 was significantly higher compared to that of UCMSCs P3 and TERT-UCMSCs P10 and P30, suggesting a more pronounced aging phenotype in non-immortalized UCMSCs (Figure 4).

### 3.5. TERT Expression

**Figure 5:**
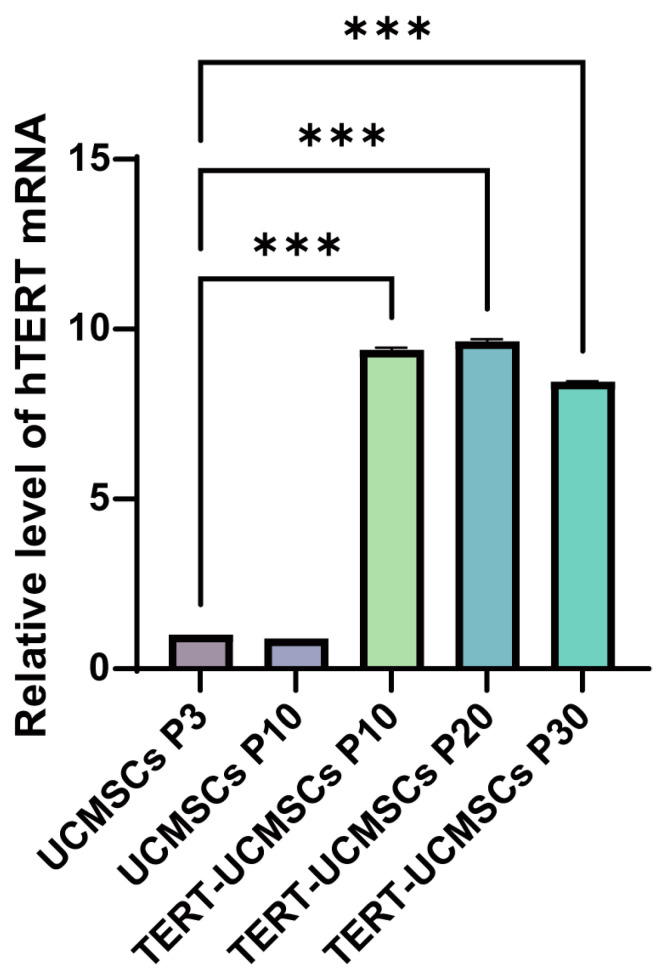
Analysis of TERT gene expression. UCMSCs P3 is used as the control group. *n* = 3, *** *p* < 0.001.

The RT-PCR results revealed a significant upregulation of the TERT gene in TERT-UCMSCs P10, P20, and P30 samples (Figure 5).

### 3.6. Growth Kinetics and PDT

**Figure 6:**
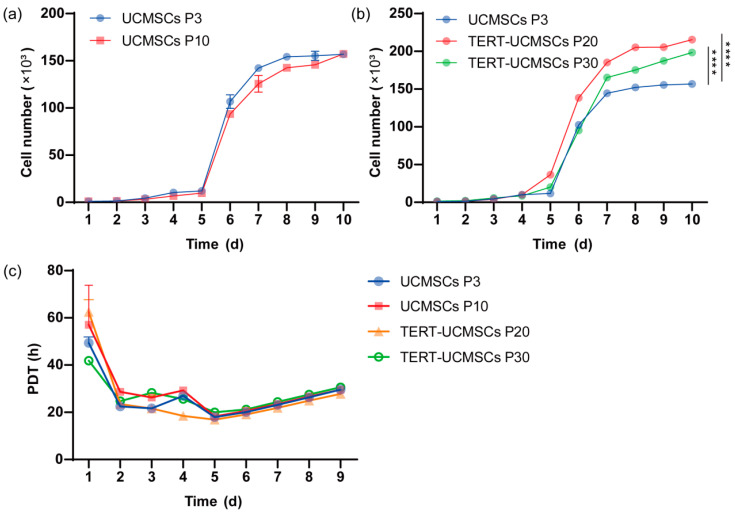
(**a**) Growth curve of UCMSCs P3 and P10; (**b**) Growth curve of UCMSCs P3, TERT-UCMSCs P20, and P30; (**c**) PDT of UCMSCs P3, P10, TERT-UCMSCs P20, and P30. *n* = 4, **** *p* < 0.0001.

The proliferation rate was assessed using primary UCMSCs P3, P10, transgenic P20, and P30 TERT-UCMSCs (Figure 6a,b). All four cell types exhibited typical S-shape with distinct phases including lag, log, and plateau periods. However, the logarithmic growth phase of transgenic UCMSCs at passage 30 showed slightly inferior performance compared to P20 TERT-UCMSCs, possibly attributed to enhanced TERT gene expression in cells at passage 20 during this stage. By the 10th day, all four cell types experienced a decline in growth rate entering a plateau phase. TERT-UCMSCs P20 had a significantly lower PDT as compared to the other passages (Figure 6c, Table S4). 

### 3.7. EdU-Azide 488 and Hoechst 33342/PI Staining

**Figure 7:**
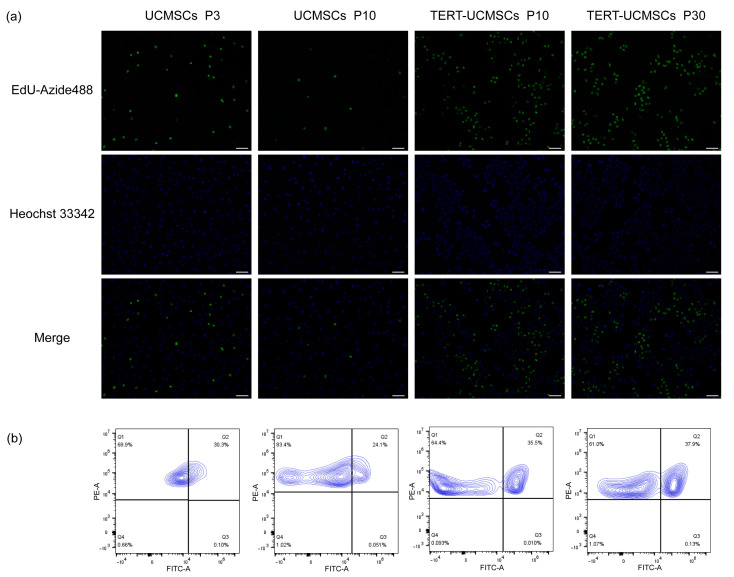
(**a**) Fluorescence results of UCMSCs P3 and P10 and TERT-UCMSCs P10 and P30 incubated with Edu working solution for 2 h then stained with EdU-Azide 488 and Hoechst 33342 (scale bar = 100 μm, *n* = 3). (**b**) Flow cytometry results of EdU-Azide 488 and PI staining of flow cytometry results of EdU-Azide 488 and PI staining of the same cells (*n* = 3, level = 5%).

EdU-Azide 488 stained newly proliferated cells within 2 h of incubation, and Hoechst 33342 was used to stain the nuclei. In UCMSCs P3, the cell proliferation rate was higher than that in UCMSCs at P10. However, the proliferation rate of TERT-UCMSCs P30 was faster than that of the other two, demonstrating that primary UCMSCs gradually undergo senescence with increasing passage numbers, and their proliferation ability was significantly improved after transfection with the TERT gene (Figure 7). The percentage of FITC-positive cells (EdU-Azide 488) in P30 was higher compared to P3, indicating an accelerated growth rate in P30 from day 7 to day 9.

### 3.8. Osteogenic, Lipogenic, and Chondrogenic Differentiation

**Figure 8:**
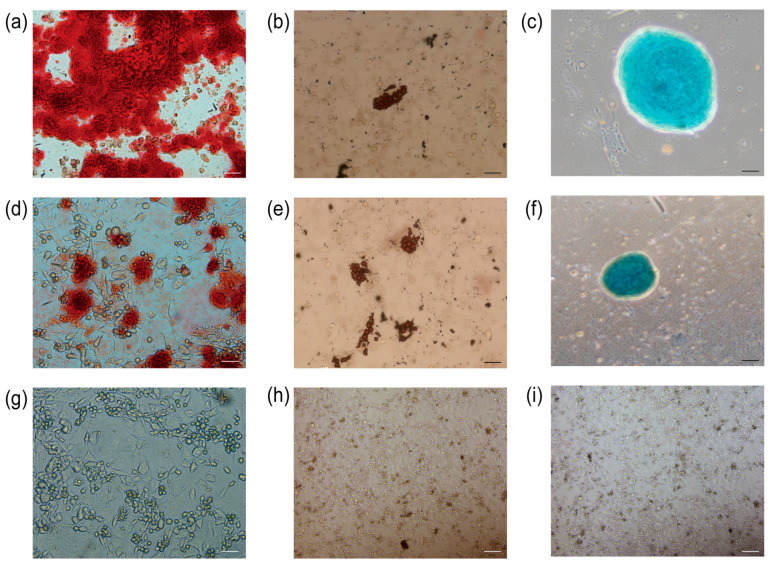
Staining results for differentiation of UCMSCs P3 and TERT-UCMSCs P30. (**a**) Osteogenic differentiation of UCMSCs P3 (scale bar = 50 μm); (**b**) Adipogenic differentiation of UCMSCs P3 (scale bar= 200 μm); (**c**) Chondrogenic differentiation of UCMSCs P3 (scale bar = 100 μm); (**d**) Osteogenic differentiation of TERT-UCMSCs P30 (scale bar= 50 μm); (**e**) Adipogenic differentiation of TERT-UCMSCs P30 (scale bar= 200 μm); (**f**) Chondrogenic differentiation of TERT-UCMSCs P30 (scale bar= 200 μm); (**g**) Negative control of UCMSCs P30 osteogenic negative control (scale bar = 50 μm); (**h**) Negative control of UCMSCs P30 adipogenic differentiation (scale bar = 200 μm); (**i**) Negative control of UCMSCs P30 chondrogenic differentiation (scale bar = 200 μm).

The primary UCMSCs and TERT-UCMSCs P30 cells were induced to differentiate into three lineages (*n* = 3). Following 2–3 weeks of induction, the induced cells were subjected to specific staining, revealing their potential to differentiate into adipocytes, chondrocytes, and osteoblasts. Adipocyte differentiation was characterized by the presence of oil droplets on day 10, which could be visualized using oil red O staining and became more prominent by day 15. Osteoblastic morphological changes were observed on day 8 and became evident after 21 days, with calcium nodules being detectable through alizarin red staining. Chondrocyte formation occurred on day 15, and cartilage could be stained using alcian blue (Figure 8).

### 3.9. Soft Agar Tumorigenicity Test

**Figure 9:**
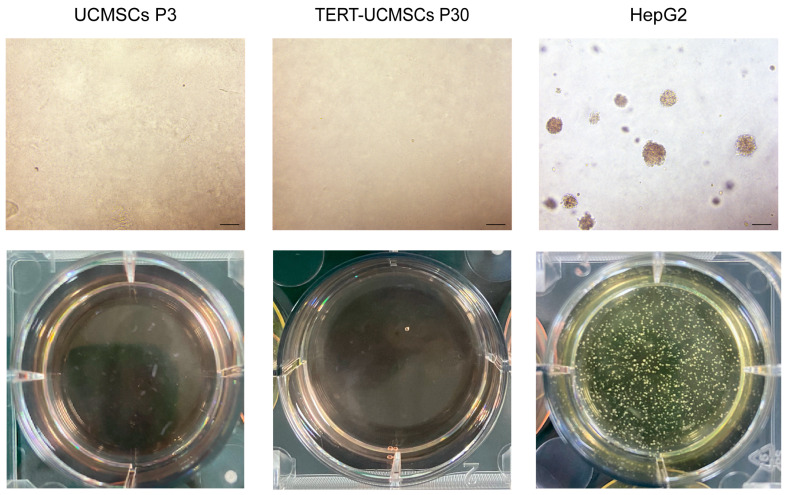
Soft agar colony formation of UCMSCs P3, TERT-UCMSCs P30, and positive control HepG2. Scale bar = 200 μm, *n* = 3.

The tumorigenicity of cells can be assessed through a soft agar assay, as normal cells are unable to grow in this medium. Primary UCMSCs and TERT-UCMSCs at passage 30 were compared to the positive control group HepG2, which was cultured in soft agar. After 15 days, colony formation was observed in the HepG2 Petri dishes, but no colonies were detected during the culturing periods of P3 and P30 UCMSCs (Figure 9).

## 4. Discussion

Mesenchymal stem cells have gained increasing prominence in regenerative medicine, immune-related disease treatment, and other fields over time. Their low rejection rate and compatibility make them suitable for allogeneic transplantation. Mesenchymal stem cells can be derived from various sources, and we chose to isolate MSCs from sheep umbilical cord Wharton’s jelly for our investigation.

Sheep umbilical cord mesenchymal stem cells were characterized according to the ability of adherent growth, surface marker expression, and differentiation potential recommended by the International Society for Cellular Therapy (ISCT) [32]. The UCMSCs were successfully isolated and met the ISCT criteria. 

However, the limited passage times and lifespan of MSCs pose challenges for large-scale production, leading to high costs. The same issue also arises in sheep. Senescence typically occurs after approximately 10 generations in sheep, characterized by increased β-galactosidase content [33], as excessive cell division and proliferation lead to telomere shortening, ultimately affecting lifespan. This issue is more pronounced in vitro due to the absence of a stable source for new cell supplementation. In vivo, MSCs benefit from a consistent iterative source and microenvironment. Nevertheless, ensuring quality and safety with each batch of supplemented cells remains difficult during in vitro culture. Extensive screening and testing are required to ensure reproducible results. Therefore, investigating methodologies to enhance the longevity of MSCs while preserving their stem cell characteristics is imperative to reduce treatment expenses and obviate the need for frequent cellular extraction from donor tissues. The objective of this study was to obtain immortalized mesenchymal stem cells for mass production and assess the capacity of human telomerase reverse transcriptase (TERT) transduced MSCs to maintain their pre-immortalized cell proliferation, differentiation ability, and surface antigen expression profile. 

Previous studies have shown that bone marrow mesenchymal stem cells (BMMSCs) and adipose-derived mesenchymal stem cells (ADMSCs) can achieve immortalization through TERT gene transfer [34], with the TERT gene effectively extending cellular lifespan across various species [35,36,37]. Therefore, we opted to transfer the TERT gene into UCMSCs to achieve immortalization.

Gene transfer techniques include liposome transfection [38], electrotransfection [39], and viral infection [40], with lentivirus being employed in our study for TERT gene transfer into UCMSCs, followed by puromycin screening. The results demonstrated that introducing the human TERT gene induced telomerase expression in MSCs and inhibited cellular senescence. Cell proliferation assays and EdU fluorescence staining showed that TERT-UCMSCs exhibited enhanced proliferative capacity compared to non-transgenic cells, possibly due to the ectopic expression of telomerase, which decelerates telomere shortening [41]. A flow cytometry analysis indicated that transgenic UCMSCs maintained consistent surface marker profiles with primary cells. Furthermore, successful passage through three-line induction testing under a conditional culture medium induction confirmed that immortalized cells retained pluripotency.

Although lentiviral vectors exhibit high efficiency in expressing foreign genes [42], their integration into the cell genome poses potential risks of proto-oncogene activation [43]. We employed the soft agar colony forming assay to investigate the potential tumorigenicity of transgenic UCMSCs. After one month of experimentation, it was determined that the 30th-generation cells of TERT-MSCs did not exhibit tumorigenic properties and were deemed safe. Furthermore, in the β-galactosidase activity test, UCMSCs at the 10th generation displayed evident staining ability, whereas TERT-UCMSCs at the 30th generation maintained a diminished level of staining intensity, exhibiting a normal state of growth. Producing these cells in large quantities establishes them as a novel source for animal cellular therapy.

## 5. Conclusions

MSCs with both proliferation and differentiation potential can be generated from umbilical cord tissue harvested from pregnant sheep. After the telomerase reverse transcriptase gene transfer, the transgenic mesenchymal stem cells (TERT-UCMSCs) were capable of undergoing over 30 passages, showing the same surface antigen expression, multilineage differentiation potential and higher proliferation ability with UCMSCs, and do not have tumorigenicity, which is a suitable source of immortalized mesenchymal stem cells.

## Figures and Tables

**Table 1:** Primer sequences for qPCR.

Gene	Primer Sequence (5′–3′)	Length (bp)
*Homo sapiens* TERT	Forward: CAGGAGCTCACGTGGAAGAT Reverse: GTAGGCGCCCATCAGCC	141
GAPDH	Forward: GGCGTGAACCACGAGAAGTATAA Reverse: CCCTCCACGATGCCAAAGT	119

**Table 2:** The expression of surface markers of mesenchymal stem cells from sheep umbilical cord. (*n* = 3).

Cell Passage Times	FITC	Markers (%)				
		CD14	CD34	CD44	CD45	CD105
UCMSCs P3	+	0.67	4.39	99.31	9.64	98.60
	−	99.33	95.61	0.69	90.36	1.40
UCMSCs P10	+	0.44	1.65	97.51	0.40	99.14
	−	99.56	98.35	2.49	99.60	0.86
TERT-UCMSCs P10	+	0.25	0.20	99.68	0.51	99.56
	−	99.75	99.80	0.32	99.49	0.44
TERT-UCMSCs P20	+	0.12	0.13	99.45	0.92	99.33
	−	99.88	99.87	0.55	99.08	0.67
TERT-UCMSCs P30	+	0.15	0.40	98.44	1.09	99.73
	−	99.85	99.60	1.56	98.9	0.27

## Data Availability

Available data are presented in the manuscript.

## Supplementary Materials

The following supporting information can be downloaded at: https://www.mdpi.com/article/10.3390/biology13070551/s1, Table S1: Components of cell osteogenic differentiation medium; Table S2: Components of cell adipogenic differentiation medium (A: induction medium; B: maintenance medium); Table S3: Components of cell chondrogenic differentiation medium; Table S4: PDT (h) data of UCMSCs P3, P10, TERT-UCMSCs P20 and P30.

## Abbreviations

MSCs: Mesenchymal stem cells; UCMSCs: Umbilical cord MSCs; TERT: Telomerase reverse transcriptase gene; ARF: Acute renal failure; IVD: Intervertebral disc degeneration; HPV: Human papillomavirus; SV40: Simian virus 40; ANOVA: Analysis of variance; BMMSCs: Bone marrow mesenchymal stem cells; ADMSCs: Adipose-derived mesenchymal stem cells; P3: Cells at passage 3; P10: Cells at passage 10; P20: Cells at passage 20; P30: Cells at passage 30.
