# Supplementary material for: Immortalization of Mesenchymal Stem Cell Lines from Sheep Umbilical Cord Tissue
Source: Biology (Basel). 2024 Jul 22;13(7):551. doi: 10.3390/biology13070551 (PMC11274198; doi:10.3390/biology13070551)
Supplement: Supplementary file 1 [file biology-13-00551-s001.zip › biology-3082599-supplementary.pdf]

Table S1. Components of cell osteogenic differentiation medium.

Components	Volume
Basal Medium(DMEM/F12=1:1)	43.395mL
Fetal Bovine Serum	5mL
Penicillin-Streptomycin	0.5mL
Glutamine	0.5mL
Ascorbate	0.1mL
β-Glycerophosphate	0.5mL
Dexamethasone	5μL
**Total**	**50mL**

Table S2. Components of cell adipogenic differentiation medium. (A: induction medium; B: maintenance medium.)

Components (A)	Volume
Basal Medium(DMEM/F12=1:1)	43.75mL
Fetal Bovine Serum	5mL
Penicillin-Streptomycin	0.5mL
Glutamine	0.5mL
Insulin	0.1mL
IBMX	50μL
Rosiglitazone	50μL
Dexamethasone	50μL
**Total**	**50mL**
**Components (B)**	**Volume**
Basal Medium(DMEM/F12=1:1)	43.9mL
Fetal Bovine Serum	5mL
Penicillin-Streptomycin	0.5mL
Glutamine	0.5mL
Insulin	0.1mL
**Total**	**50mL**

Table S3. Components of cell chondrogenic differentiation medium.

Components	Volume
Basal Medium(DMEM/F12=1:1)	48.745ml
Dexamethasone	5μL
Ascorbate	0.15mL
ITS+Supplement	0.5ml
Sodium Pyruvate	50μL
Proline	50μL
TGF-β3	0.5ml
**Total**	**50mL**

Table S4. PDT (h) data of UCMSCs P3 ,P10, TERT-UCMSCs P20 and P30.

Days	MSCs P3	MSCs P10	TERT-UCMSCs P20	TERT-UCMSCs P30
**1**	49.37	57.00	62.50	41.83

**2**	22.51	28.68	23.49	24.79
**3**	21.67	26.30	21.66	28.31
**4**	26.93	29.28	18.49	25.61
**5**	17.97	18.33	16.87	20.04
**6**	20.07	20.66	19.12	21.22
**7**	23.17	23.48	21.87	24.46
**8**	26.37	26.71	24.99	27.57
**9**	29.62	29.60	27.87	30.65
